# Circulating microRNA dysregulation in hypertrophic cardiomyopathy, arrhythmogenic cardiomyopathy, and dilated cardiomyopathy: a systematic review

**DOI:** 10.1186/s13643-026-03246-2

**Published:** 2026-06-26

**Authors:** Laurens Léger, Sabina Ferron, Nele S. Pauwels, Franne Lingier, Alba Rodriguez-Muñoz, Mora Murri-Pierri, Jolanda van Hengel, Martina Calore

**Affiliations:** 1 Medical Cell Biology Research Group, Department of Human Structure and Repair, Faculty of Medicine and Health Sciences, Ghent University, Ghent, Belgium; 2 Department of Biology, Padova University, Padua, Italy; 3 Knowledge Centre for Health Ghent, Ghent University Hospital, Ghent, Belgium; 4 Department of Endocrinology and Nutrition, Virgen de la Victoria University Hospital and Instituto de Investigación Biomédica de Málaga y Plataforma en Nanomedicina-IBIMA Plataforma BIONAND, Malaga, Spain; 5 Faculty of Health Sciences, University of Malaga, Malaga, Spain; 6 CIBER Fisiopatología de La Obesidad y Nutrición (CIBEROBN), Instituto de Salud Carlos III, Malaga, Spain; 7 Department of Cardiology and Cardiovascular Surgery, Virgen de La Victoria University Hospital and Instituto de Investigación Biomédica de Málaga y Plataforma en Nanomedicina-IBIMA Plataforma BIONAND, Malaga, Spain; 8 Department of Cardiology, Faculty of Health, Medicine and Life Sciences, Maastricht University, Maastricht, The Netherlands

**Keywords:** MiRNAs, Inherited cardiomyopathy, Biomarkers, Hypertrophic cardiomyopathy, Arrhythmogenic cardiomyopathy, Dilated cardiomyopathy, Heart failure

## Abstract

Hypertrophic cardiomyopathy (HCM), arrhythmogenic cardiomyopathy (ACM) and dilated cardiomyopathy (DCM) represent the most frequent structural heart disorders. Due to the complexity of their diagnosis and the frequent occurrence of sudden cardiac death as the first manifestation, these diseases impose a relevant socio-economic burden, highlighting the urgent need for novel disease-specific biomarkers. Here, we assess the potential of microRNAs (miRNAs) as non-invasive predictors for HCM, ACM, and DCM. Electronic databases were systematically searched using relevant keywords pertaining to the disorders and miRNAs. To minimize spurious associations, only studies reporting the area under the curve (AUC) and including healthy controls were taken into account, resulting in the selection of 19 papers for further analyses. miR-29a-3p, miR-185-5p, and miR-454-3p were found overexpressed in HCM, ACM, and DCM, respectively, across different studies. Assessment of the correlation between circulating miRNA levels and cardiac features or genetic background failed to detect consistent profiles among different studies. Thus, further investigations employing consistent approaches and larger cohorts are required to validate the clinical utility of circulating miRNAs as non-invasive biomarkers for these structural cardiomyopathies.

**Systematic review registration** PROSPERO CRD42023330236.

**Supplementary Information:**

The online version contains supplementary material available at https://doi.org/10.1186/s13643-026-03246-2.

## Introduction

Cardiovascular disease (CVD) is one of the leading causes for death, accounting for up to a third of all deceased worldwide. Within CVD, cardiomyopathy is defined as a myocardial disorder with a structurally and functionally abnormal heart without underlying coronary artery disease, hypertension, valvular disease, and congenital heart disease [1]. The most frequent forms of structural cardiomyopathies, which are primarily characterized by alterations in the heart muscle tissue, are hypertrophic cardiomyopathy (HCM), arrhythmogenic cardiomyopathy (ACM), and dilated cardiomyopathy (DCM).

HCM is defined by otherwise unexplained myocardial hypertrophy, typically diagnosed through increased ventricular thickness or mass in the absence of hemodynamic stress or systemic diseases such as amyloidosis or glycogen storage disorders [1]. HCM can be differentiated into hypertrophic obstructive cardiomyopathy (HOCM) or hypertrophic nonobstructive cardiomyopathy (HNCM) depending on the presence of left ventricular outflow tract obstruction [2].

ACM is defined by fibrous or fibro-fatty myocardial replacement affecting the right, left, or both ventricles, often resulting in wall thinning, which, especially in the later stages, may be misdiagnosed as DCM, ventricular arrhythmias, and syncope [3]. Originally identified as arrhythmogenic right ventricular cardiomyopathy (ARVC), the broader term ACM is currently used due to the frequent bi-ventricular or left-dominant involvement [4, 5].

DCM, in its genetic or idiopathic form, is characterized by left ventricular dilatation and systolic dysfunction in the absence of abnormal loading conditions (hypertension, valvular disease) or coronary artery disease sufficient to explain the global impairment. Right ventricular involvement may occur, but is not required for diagnosis [1]. In most cases, all of these structural cardiomyopathies are inherited as an autosomal dominant trait with significant phenotypic variability, even among carriers of the same pathogenic variant [1–3].

In these disorders, sudden death may be the first manifestation, underlining the need for early diagnosis and the importance of cascade screening for at-risk relatives. Diagnostic challenges arise from the broad phenotypic variability, borderline presentations, and the need for invasive interventions (e.g., endomyocardial biopsy), or postmortem assessments [6]. Overall, cardiomyopathies represent a major global health burden accounting for a substantial proportion of cardiac transplants, defibrillator implantations and circulatory assistance [7]. Given these diagnostic limitations, there is a strong need for novel, disease-specific biomarkers.

Besides being sensitive and specific for a given disease, a good biomarker should be stable in the biological fluids, easy and cost-effective to measure using standard laboratory techniques or minimally invasive procedures, and its measurement should be reproducible across different laboratories and assay methods [8]. Circulating microRNAs (miRNAs) have gained attention as potential biomarkers in inherited cardiomyopathies. miRNAs are small (~ 22 nucleotides long) single-stranded evolutionarily conserved non-coding RNA molecules. They have an important role in regulating gene expression post-transcriptionally, by degrading their target mRNA or by blocking translation, in different pathophysiological conditions and in cellular homeostasis [9]. In cardiomyopathies, several miRNAs regulate pathways involved in hypertrophy, fibrosis, electrical conduction, and cardiomyocyte survival, providing a direct mechanistic link between miRNA dysregulation and the structural and functional abnormalities observed in these inherited cardiomyopathies (HCM, ACM, and DCM) [10–12].

Interestingly, although primarily intracellular, miRNAs can also be secreted into extracellular fluids and transported to target cells by vesicles such as exosomes, or by binding to Argonaute, remaining stable and measurable. Because they can be measured in peripheral blood without requiring invasive procedures, circulating miRNAs represent attractive non-invasive biomarkers with the potential to complement current diagnostic approaches in inherited cardiomyopathies. This makes extracellular miRNAs appealing biomarkers for different conditions, including inherited cardiomyopathies [9]. Distinct circulating miRNAs have been linked to specific heart failure phenotypes (HFrEF vs. HFpEF), etiologies (ischemic vs. non-ischemic), disease stages (acute vs. chronic), and therapeutic responses, such as to cardiac resynchronization therapy (CRT) and left ventricular assist devices (LVAD) [13–15], supporting their potential diagnostic and prognostic value.

A preliminary search in several datasets highlighted that while several reviews have explored miRNAs in cardiomyopathies, none has fully addressed the scope we aim to cover. For example, a systematic review published in 2021 examined miRNA profiles solely in patients with HCM [16], whereas a recent narrative review considered the three major inherited cardiomyopathies, but did not focus specifically on circulating miRNAs as non-invasive biomarkers in humans [17]. Other systematic reviews have recently investigated circulating miRNAs in HCM for predicting myocardial fibrosis [18], yet none have comprehensively analyzed HCM, DCM, and ACM in a single systematic review, integrating the potential of circulating biomarkers with pathogenic gene variants and diagnostic applications. To our knowledge, this is the first systematic review to evaluate circulating miRNAs as potential non-invasive biomarkers across these three major inherited cardiomyopathies, providing a unified and up-to-date synthesis of the evidence.

Given the difficulties in diagnosis and the high risk of sudden cardiac death related to the three major inherited cardiomyopathies, this review aims to systematically assess the diagnostic potential of circulating miRNAs in the three main types of structural cardiomyopathies (i.e., HCM, ACM and DCM), to highlight possible promising novel biomarkers useful for diagnosing these life-threatening afflictions.

## Methods

This review was reported following the 2020 Preferred Reporting Items for Systematic Reviews and Meta-analysis (PRISMA) guidelines [19] and the protocol was registered in and made available via PROSPERO [20], the International Prospective Register of Systematic Reviews (https://www.crd.york.ac.uk/prospero/ Identifier: CRD42023330236). The following databases were interrogated for original studies: MEDLINE (via the PubMed interface), Embase (via Embase.com interface), SCOPUS, CENTRAL and Web of Science. For the main concepts of this systematic review, being the different cardiomyopathies and circulating miRNAs, relevant subject headings (such as MeSH and Emtree terms) were selected and combined with synonyms searched in titles, abstracts and author keywords. These search strategies were then translated to the other databases’ syntax. The full search terms are given in Supplementary file 1. The search strategy adheres to the Peer Review of Electronic Search Strategies (PRESS) criteria for electronic search strategies as stated in the 2015 Guideline Statement [21]. Databases were searched in September 2023 and last searched on September 5th 2024. To ensure complete coverage, a time overlap was implemented between the two searches. Only articles in English were included. No publication period restriction was imposed. The objective of the selection process was to identify studies providing empirical evidence of differential circulating miRNA levels between cases and comparators. Therefore, study selection focused on both design characteristics (presence of a comparator group, human samples, and reported diagnostic metrics) and analytical approach (validation step, ROC analysis) used by the authors.

### Study selection

For our study population, we included patients of all ages, genders, and ethnicities with diagnosed forms of cardiomyopathies (HCM, DCM, ACM). Inherited cardiomyopathy was defined as the presence of a pathogenic or likely pathogenic genetic variant, a positive family history, or clinical diagnostic criteria consistent with an inherited form. Genetic forms were therefore classified as inherited. In this literature study, we compared circulating miRNA profiles between different patients affected with inherited structural cardiomyopathies (HCM, ACM, DCM). Therefore, in vivo animal/in vitro studies were disregarded. Other types of RNA molecules, such as long non-coding RNAs or small nucleolar RNAs, were beyond the scope of this review, thus excluded. Reviews, systematic reviews, meta-analyses and grey literature were excluded as well. Grey literature was excluded to ensure methodological robustness and to include only studies that had undergone peer review. This decision aimed to reduce variability in study quality and increase the reliability of circulating miRNA measurements. Eligible study designs included case–control and cross-sectional studies that reported a comparator group. Case–control studies were included because they represent the standard design for early biomarker discovery, allowing clear comparison of affected individuals with appropriate control groups. Case reports and case series were excluded because they do not allow comparative assessment of circulating miRNA levels. Studies were eligible if they included healthy controls (i.e., non-affected individuals), or both healthy controls and disease controls (i.e., patients affected with other cardiac disorders).

Title/abstract and full-text screening were performed independently by two reviewers (LL and MC) following the Cochrane guidelines to lower the risk of bias with a two-step screening process using the Rayyan screening platform to maintain traceability of decisions [22]. Reviewers were blinded to each other’s selections during the initial screening. Disagreements were first discussed to reach consensus; if consensus was not achieved, a third reviewer (JvH) was consulted to resolve the conflict through discussion with all parties.

### Data extraction

Data extraction was conducted according to four conceptual domains:

(1) Characteristics of the study: authors, year of publication, study design, and stated research objectives; (2) characteristics of participants: sample size, demographics, cardiomyopathy subtype, diagnostic criteria used, and definition of inherited disease (genetic variant, family history, or clinical criteria), along with comparator group; (3) laboratory and technical parameters: biological specimen (plasma, serum, PBMCs), profiling platform (RT-qPCR, microarray, NGS, normalization step); (4) diagnostic and biological outputs: differentially expressed miRNAs, direction of change, ROC/AUC and related diagnostic metrics, form of experimental validation.

Studies were required to report ROC/AUC values to ensure comparability of diagnostic test accuracy across biomarkers. ROC/AUC was not used to exclude studies based on performance, but as a standardized diagnostic metric necessary for evaluating the diagnostic potential of circulating miRNAs. We acknowledge that requiring ROC/AUC reporting may introduce selection toward exploratory studies reporting diagnostic performance. This risk was considered acceptable because standardized diagnostic metrics were essential for comparing biomarkers. Nevertheless, all diagnostic estimates should be interpreted cautiously in light of the limited validation and methodological heterogeneity across studies.

### Risk of bias and quality assessment

To score the quality of the publications included in this study, the Quality Assessment of Diagnostic Accuracy Studies-2 (QUADAS-2) tool was used [23]. Each article was assessed by two researchers on the following four domains: Patient selection, Index test(s), Reference standard and Flow and Timing. Each criterion was scored with “high”, “low” or “unclear” risk of bias. The QUADAS-2 tool was applied without structural modification. One question required contextual interpretation to ensure applicability to circulating miRNA profiling studies. This approach preserved the original framework of the tool while allowing an appropriate assessment of risk of bias. Two researchers (LL and MC) independently and blindly applied the checklist to the selected articles. Any disagreements between reviewers were resolved through discussion to reach consensus. If consensus could not be achieved, a third reviewer (JvH) was consulted to adjudicate and resolve the disagreement.

Following standard approaches for systematic reviews of diagnostic biomarkers, studies were not excluded based solely on a low QUADAS-2 score. Quality assessment was performed to inform the interpretation of the evidence and to describe methodological limitations, but not to exclude studies based on quality. The exclusion of low-quality studies would have created a risk of selection bias and reduced the comprehensiveness of the review, especially in view of the limited number of studies available within this area.

## Results

### Literature search

**Fig. 1:**
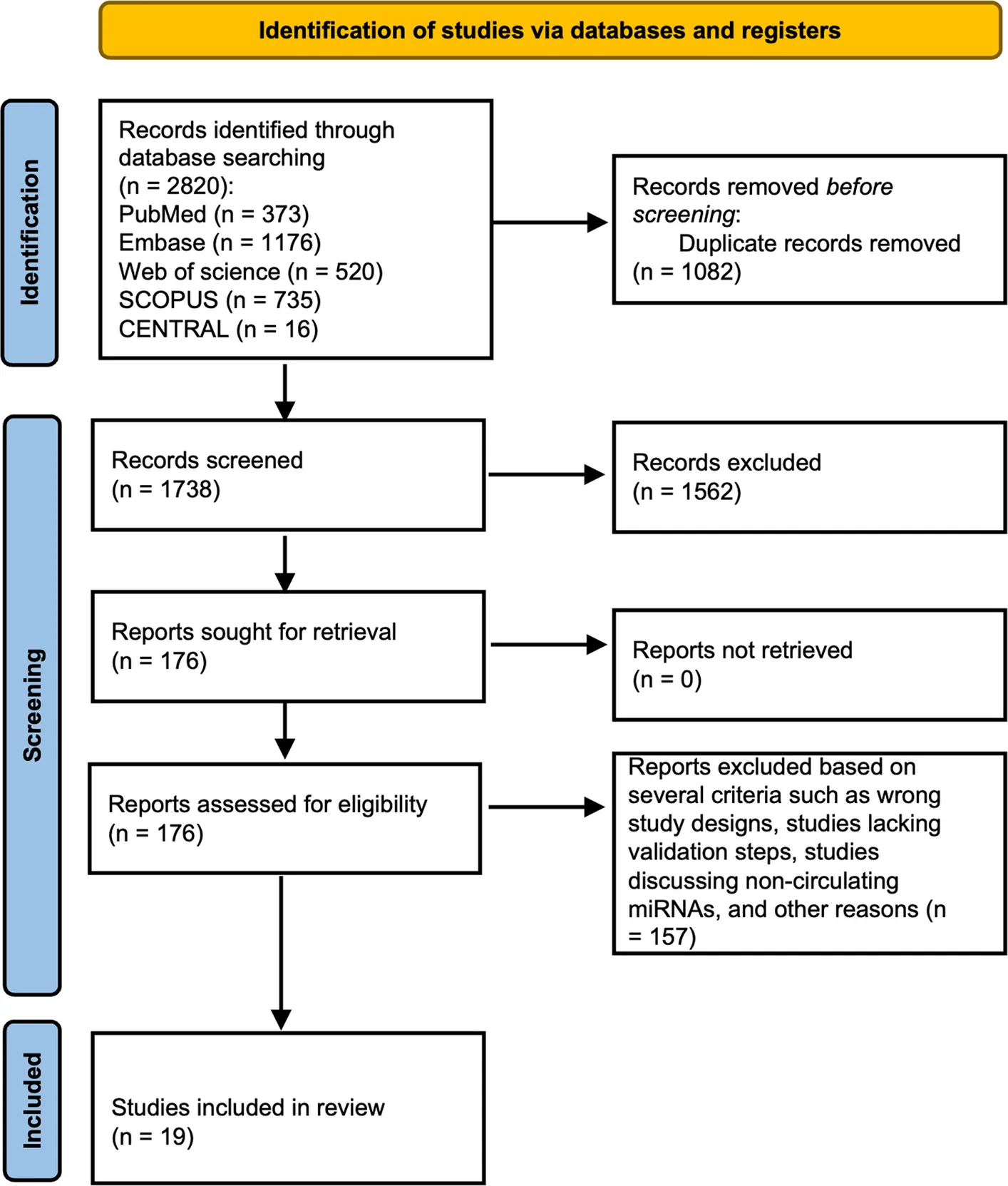
PRISMA flow diagram of studies on circulating miRNAs in HCM, ACM, and DCM

The study selection is presented as the PRISMA Flow Diagram (Fig. 1) including the number of records searched at two timepoints and last searched on September 5th 2024.

### Quality assessment

**Fig. 2:**
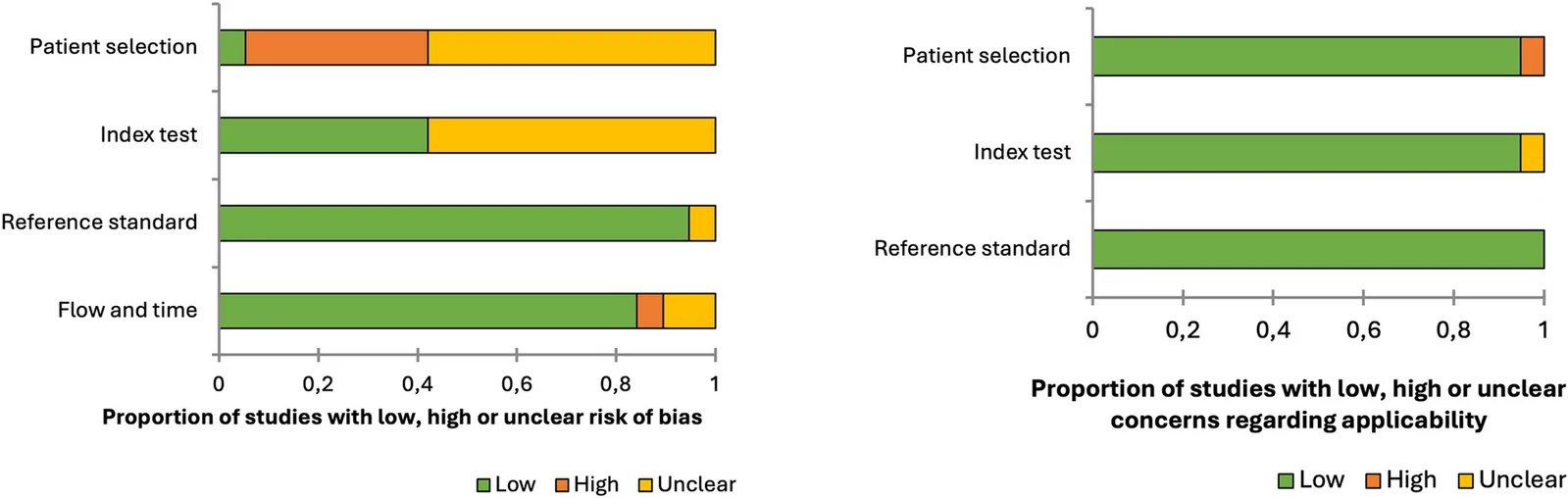
Risk of bias assessment of the included studies using the QUADAS-2 tool

Risk of bias and quality assessment was performed using the QUADAS-2 checklist. The included studies were scored on the following four domains: Patient selection, Index test, Reference standard and Flow and Timing. A summarizing graph can be found in Fig. 2. The highest risk of bias was identified in the patient selection domain, as it was often unclear if a consecutive or random sample of patients was enrolled. Furthermore, a case–control study design was often employed. About half of the studies had unclear risk of bias for the index test, as the threshold was often not pre-specified. The majority of studies had no concerns regarding applicability. The full list for risk of bias and quality appraisal is given in Supplementary Fig. S1.

### Characteristics of included studies

In this section, the term ‘inherited cardiomyopathies’ refers collectively to HCM, ACM, and DCM. A total of nineteen studies met the inclusion criteria and were included in this review, covering the three major inherited cardiomyopathies: HCM, ACM, and DCM. The studies included were conducted on 260 HCM (78 defined as HOCM and 31 as HNCM) patients vs 177 healthy controls [24–30]; 200 ACM patients vs 140 healthy controls [31–34]; 428 DCM patients vs 369 healthy controls (83 pediatric DCM patients vs 65 healthy controls) [35–42]. Most studies used RT-qPCR (*n* = 9) [24–27, 29, 36, 37, 39–41] or microarray (*n* = 7) [30–35, 38] to assess the population of miRNAs, while only few studies (*n* = 3) [26, 28, 42] were based on next generation sequencing. Fourteen studies used plasma [25–29, 31–37, 40, 41], four studies used serum [24, 38, 39, 42] and one used peripheral blood mononuclear cells (PBMC) [30].

### Circulating miRNAs dysregulated in HCM

A total of seven studies focused on HCM [24–30] (Table 1). Of these, three discriminated between HOCM and HNCM [24, 29, 30], one focused only on HOCM cases [25], while three included both HOCM and HNCM patients, without distinction [26–28]. All of the studies included healthy controls, while two also analyzed patients with cardiomyopathies of different etiology to assess miRNA specificity, including aortic stenosis (AS) [26, 27] and, in one study, senile cardiac amyloidosis (SCA) [27]. Five studies investigated miRNA levels in plasma, while one used serum instead [24] and one PBMC [30]. Quantification was conducted through RT-qPCR for selected miRNAs [24, 25, 27, 29], next generation sequencing (NGS) [26, 28], or microarray [30].

**Table 1:** Table summarizing the studies on circulating miRNAs in HCM patients

Ref	Controls	Primary screening	Starting Material	Interrogated miRNAs	DE miRNAs	AUC
[28]	Healthy controls	NGS	Plasma	1128	↑ miR-19a-3p, miR-20b-5p, miR-29b-3p, miR-126-5p, miR-144-3p, miR-4732-5p ↓ miR-182-5p	0.9091 0.8434 0.9489 0.7409 0.798 0.8091 0.7879
[27]	Healthy controls, patients with AS	RT-qPCR	Plasma	21	↑ miR-27a, miR-199a-5p, miR-26a, miR-145, miR-133a, miR-143, miR-199a-3p, miR-29a	0.8939 0.8323 0.7792 0.8130 0.7114 0.8877 0.8387 0.8365
[24]	Healthy controls, patients with cardiac hypertrophy due to AS and patients with SCA	RT-qPCR	Serum	8	↑ miR-29a (HOCM) ↓ miR-155	0.7045 0.7478
[25]	Healthy controls	RT-qPCR	Plasma	11	↑ miR-221 (HOCM)	0.764
[26]	Healthy controls without *MYH7* variants	Small RNA sequencing	Plasma	n.a	↑ miR-499a-5p	0.95
[29]	Healthy controls	RT-qPCR	Plasma	1	↑ miR-146a	0.669
[30]	Healthy controls	Custom CeRNA Human Gene Expression Microarray	PBMC	2798	↑ miR-924, miR-98, miR-1	0.829 0.866 0.866

*DE miRNAs* differently express microRNAs, *AUC* area under the curve, *AS* aortic stenosis, *SCA* senile cardiac amyloidosis, *n.a.* not available, *PBMC* peripheral blood mononuclear cells

A total of twenty circulating miRNAs were found significantly upregulated in HCM patients compared to healthy controls or patients with AS/SCA, when included (Table 1) [24, 27]. Of these, miR-29a was validated in two independent studies, including one specifically in HOCM patients [24, 27]. In contrast, only a few miRNAs were found downregulated [24, 28]. Comparisons between HOCM and HNCM highlighted miR-146a and miR-221 as upregulated in HOCM cohorts [25, 29]. Three miRNAs (miR-19a-3p, miR-29b-3p, miR-499a-5p) showed high diagnostic potential (AUC > 0.9), although they were investigated in HCM patients without discriminating between HOCM and HNCM [26, 28].

### Circulating miRNAs dysregulated in ACM

Four papers investigated miRNA expression in the plasma of ACM patients, all including healthy controls (Table 2) [31–34]. Moreover, ACM patients categorized as borderline or possible ARVC were also considered in one study [31], individuals with idiopathic ventricular tachycardia in two studies [31, 32], and unaffected family members of ACM probands carrying a desmosomal pathogenic variant, along with patients with HCM, DCM, Brugada syndrome, or myocarditis [34]. miRNA expression was assessed using RT-PCR-based arrays including 84 [31, 34], 377 [32], or 754 miRNAs [33], with one study integrating small RNA sequencing in blood [34].

**Table 2:** Table summarizing the studies on circulating miRNAs in ACM

Ref	Controls	Primary screening	Starting Material	Interrogated miRNAs	DE miRNAs	AUC
[31]	Healthy controls, Borderline or possible ARVC, patients with IVT	miScript miRNA PCR Array kit	Plasma	84	↑ miR-144-3p, miR-145-5p, miR-185-5p, miR-494	0.728 0.619 0.740 0.891
[34]	Healthy controls, non-affected family members of AC probands carrying a desmosomal pathogenic variant, HCM, DCM, BrS and myocarditis affected patients	miRNA array followed by NGS	Plasma	84	↑ miR-122-5p, miR-182-5p, miR-183-5p, ↓ miR-133a-3p, miR-133b, miR-142-3p	0.9146 0.7371 0.707 0.845 0.858 0.9692
[32]	Healthy controls	TaqMan Human microRNA array	Plasma	377	↓ miR-320a	0.69
[33]	Healthy controls	TaqMan Human microRNA assay	Plasma	754	↑ miR-185-5p	0.854

*DE miRNAs* differently express microRNAs, *AUC* area under the curve, *ARVC* Arrhythmogenic Right Ventricular Cardiomyopathy, *IVT* Idiopathic ventricular tachycardia, *BrS* Brugada syndrome

Across the studies, seven miRNAs were reported as significantly upregulated [31, 33, 34] and four downregulated [32, 34], when compared to healthy controls [31–34], or patients with idiopathic ventricular tachycardia [32] (Table 2).

Only one of those miRNAs (miR-185-5p) was found to be significantly upregulated, compared to healthy controls, in two different cohorts [31, 33].

Two miRNAs, miR-122-5p and miR-142-3p, showed high diagnostic potential (AUC > 0.9) [34] and a panel of six differentially expressed miRNAs (miR-122-5p, miR-182-5p, miR-183-5p, miR-133a-3p, miR-133b, miR-142-3p) revealed a strong discrimination between individuals with a definitive form of ACM, healthy controls, and patients with other cardiac conditions (AUC 0.9146–0.987) [34]. Three of these miRNAs (miR-122-5p, *p* < 0.001; miR-182-5p, *p* < 0.001; miR-182-3p, *p* < 0.001) were found differentially expressed also in asymptomatic carriers of pathogenic variants, suggesting a potential role in early disease diagnosis for patients with a family history [34].

### Circulating miRNAs dysregulated in DCM

Eight papers examined miRNA levels in patients with idiopathic [35–39], familial [37, 40], or pediatric DCM [41, 42] compared to healthy controls (Table 3). One of these studies compared miRNA expression also to findings from patients with inflammatory and/or virally induced myocardial disease [38]. Four groups studied plasma [35–37, 40], while two used serum [38, 39]. miRNA profiling was performed using RT-qPCR [36, 37, 39–41], arrays of varying coverage [35, 38], or NGS [42]. Across these studies, twenty miRNAs were upregulated and four were downregulated in DCM samples compared to healthy controls and other cardiopathic patients, when included (Table 3). Specifically, let-7a-5p, miR-142-3p, miR-145-5p and miR-454-3p were upregulated in familial DCM [37], while miR-194-5p, miR-454-3p, let-7f-5p, let-7 g-5p, miR-26a-5p, miR-27a-3p, miR-27b-3p, miR-126-3p, miR-142-5p, miR-143-3p were found upregulated in pediatric DCM [41, 42] (Table 3). Across studies, miR-454-3p emerged as one of the most consistently reported DCM-associated circulating miRNAs, being identified in both familial and pediatric cohorts [37, 41].

**Table 3:** Table summarizing the studies on circulating miRNAs in DCM

Ref	Controls	Primary screening	Starting material	Interrogated miRNAs	DE miRNAs	AUC
[37]	Healthy controls, iDCM with wild type *LMNA*	microRNA Panel RT-PCR	Plasma	179	↑ let-7a-5p, miR-142-3p, miR-145-5p, miR-454-3p (*LMNA* pathogenic variants)	0.812 0.764 0.76 0.713
[40]	Healthy controls	RT-qPCR	Plasma	5	↑ miR-423-5p	0.674
[35]	Healthy controls	Genome-wide microarray screening	Plasma	n.a	↑ miR-3135b miR-3908 miR-5571-5p	1 0.83 0.91
[36]	Healthy controls	RT-qPCR	Plasma	1	↑ miR-16	0.645
[38]	Healthy controls and patients with inflammatory and/or virally induced myocardial disease	TaqMan OpenArray	Serum	754	↑ miR-21, miR-30a-5p	0.93 1
[39]	Healthy controls	RT-qPCR	Serum	9	↓ miR-26a-5p miR-30c-5p miR-126-5p miR-126-3p	0.7146 0.7176 0.6876 0.7198
[41]	Healthy controls	RT-qPCR	Plasma	5	↑ miR-194-5p miR-454-3p (pediatric DCM)	0.798 1
[42]	Healthy controls	NGS	Serum	1675	↑ let-7f-5p let-7 g-5p miR-26a-5p miR-27a-3p miR-27b-3p miR-126-3p miR-142-5p miR-143-3p (pediatric DCM)	0.89 0.92 0.92 0.73 0.92 0.95 0.98 0.92

*DE miRNAs* differently express microRNAs, *AUC* area under the curve, *LMNA* Lamin A/C gene, *iDCM* idiopathic dilated cardiomyopathy, *IHD* ischemic heart disease, *n.a.* not available

Correlation analyses showed that let-7a-5p (*p* < 0.001), miR-142-3p (*p* = 0.001), miR-145-5p (*p* < 0.001), miR-454-3p (*p* = 0.003) [37] expression correlated with the presence of a LMNA pathogenic variant, also in asymptomatic individuals, while miR-5571 significantly correlated with a more severe form of DCM accordingly with New York Heart Association (NYHA III-IV) classification (*p* < 0.05) [35]. Eleven miRNAs showed high diagnostic potential (AUC > 0.9), including miR-3135b, miR-5571-5p, miR-21, miR-30a-5p, miR-454-3p, let-7 g-5p, miR-26a-5p, miR-27b-3p, miR-126-3p, miR-142-5p, and miR-143-3p. Among these miRNAs, miR-454-3p and miR-26-5p were validated in two independent studiesmiR-454-3p was consistently upregulated [37, 41], while miR-26-5p was upregulated in pediatric DCM and downregulated in DCM [39, 42].

### miRNA level and phenotype correlation

Inherited cardiomyopathies often result in cardiac remodeling, including hypertrophy and fibrosis, abnormal cardiac function, and ultimately heart failure [43]. Across the included studies, correlations between miRNA levels and phenotypic traits varied considerably, and only a few consistent patterns emerged. This systematic review identified a significant correlation between miR-144-3p (*p* = 0,003), miR-20b-5p (*p* = 0.0247) and miR-221 (*p* < 0.001) with left ventricular ejection fraction (LVEF) in both HNCM and HOCM [25, 28]. Moreover, miR-221 (*p* < 0.001 and *p* = 0.044) and miR-29a-3p (*p* = 0.005 and *p* = 0.021) exhibited positive correlations with myocardial fibrosis and hypertrophy, respectively, in HCM patients [24, 25, 27]. The latter also was significantly correlated with miR-27a (*p* = 0.032) and miR-199a-5p (*p* = 0.017) [27]. Additionally, miR-146a-5p levels were significantly increased in HOCM, but not in HNCM (*p* = 0.02) [29]. Associations in ACM and DCM were more sporadic and often study-specific. In ACM patients, only the correspondence between circulating miR-494 and ventricular arrhythmias was found (*p* < 0.05) [31]. Furthermore, plasma levels of let-7a-5p (*p* < 0.001), miR-142-3p (*p* = 0.001), miR-145-5p (*p* < 0.001) and miR-454-3p (*p* = 0.003) inversely correlated with cardiac dilation in DCM patients harboring pathogenic variants in the LMNA gene [37]. Conversely, LVEF showed positive and negative associations with let-7a-5p (*p* = 0.035) [37] and miR-126-5p (*p* = 0.007) and let-7 g-5p (*p* = 0.016) [42], respectively. In addition, miR-5571-5p correlated with a more severe form of the disease according to the NYHA classification (*p* < 0.05) [35]. The weak correlation between miRNA levels and phenotype also reflects the limited number of studies examining this relationship, as well as the inconclusive results often reported. Five studies did not conduct phenotype correlation analyses [26, 30, 33, 34, 41]. On the contrary, the remaining studies did not demonstrate any correlation [32, 38, 40].

### Circulating miRNAs and genes harboring pathogenic variants

HCM, DCM, and ACM are heterogeneous monogenic heart diseases [43]. In this systematic review, we also highlighted the relationship between genes harboring pathogenic variants found in affected patients and circulating miRNA levels. While no study successfully investigated this aspect in ACM, MYH7 pathogenic variations, frequently found in HCM patients, were associated with increased levels of miR-29a (*p* < 0.05) and miR-499-5p (*p* < 0.0001) [24, 26]. Interestingly, both miRNAs did not show the same trend in MYBPC3 variation carriers, who, on the contrary, displayed lower levels of miR-155-5p (*p* < 0.05) [24]. As far as DCM is concerned, let-7a-5p (*p* < 0.001), miR-142-3p (*p* = 0.001), miR-145-5p (*p* < 0.001), miR-454-3p (*p* = 0.003) levels were higher in patients harboring a LMNA pathogenic variant, both showing disease signs or fully asymptomatic, compared to healthy patients [37]. Of note, these miRNAs were not altered in idiopathic DCM patients, suggesting a direct link between genetic alterations in the LMNA gene and the control of miRNA expression [37]. These findings support a mechanistic link between genetic variants and circulating miRNA regulation, suggesting that specific variants may modulate intracellular signaling pathways that ultimately shape the detectable circulating miRNA profile.

## Discussion

In this systematic review, a total of nineteen studies were included, of which seven comprised 260 HCM patients, four included 200 ACM patients, and eight (of which two on pediatric DCM) investigated 345 DCM patients and 83 pediatric DCM cases.

Altogether these studies identified several circulating miRNAs as promising candidates showing high diagnostic potential within their respective cohorts. These findings suggest that circulating miRNAs are capable of detecting molecular changes relevant in inherited cardiomyopathies and support their non-invasive diagnosis. However, the overlap between studies was limited, due to both heterogeneous analytical methods and profiled miRNAs. For this reason, these data represent only preliminary evidence, and further validation in independent cohorts will be essential to determine their true robustness and disease specificity.

### Diagnostic potential of circulating miRNAs

Only three miRNAs, miR-29a-3p, miR-185-5p, and miR-454-3p, were consistently found upregulated in inherited cardiomyopathies in more than one study [24, 27, 31, 33, 37, 41]. Circulating miR-29a-3p was found upregulated in HOCM but not in HNCM [24]; however, Roncarati et al. [27] also observed its overexpression in HCM samples without stratifying patients by subtype. Furthermore, a study excluded from our analysis involving a cohort of HCM patients demonstrated a positive correlation with both QRS duration and cardiac hypertrophy (*p* = 0.043) [44]. Moreover, miR-454-3p was found upregulated in both DCM and pediatric DCM (in the last case AUC = 1) [37, 41]. This is in line with the fact that these two forms share many phenotypic features. Of note, miR-454-3p was found upregulated also in the plasma of HCM patients [28]; however, that result was discarded due to inadequate diagnostic accuracy by ROC analysis.

Interestingly, seven miRNAs (miR-145-5p, miR-142-3p, miR-143-3p, miR-144-3p, miR-27a-3p, miR-26a-5p, miR-182-5p) showed consistent dysregulation in more than one cardiomyopathy. The recurrence of the same dysregulation in the three disorders suggests that these miRNAs might not be disease-specific biomarkers, but rather indicators of cardiac dysfunction. Their analysis in patients with other cardiac disorders may help assess this hypothesis. Moreover, despite not being cardiomyopathy-specific, these dysregulated circulating miRNAs may still be potentially useful to identify individuals with underlying cardiac pathology who may benefit from further diagnostic evaluation.

Most other miRNAs were found dysregulated in single investigations only. The lack of reproducibility across studies likely reflects technical and methodological bias. Indeed, the selected studies were heterogeneous in terms of biological starting material, sample preparation, and choice of control population (i.e., healthy individuals or patients with other cardiac diseases). Additional influencing factors may include the analytic approach, consisting of either targeted panels or genome-wide screening; microarray, or qPCR, and the validation system. Besides technical factors, also patient-related variables, such as sex, genetic background, disease stage, comorbidities, medical treatments and lifestyle may have influenced the outcome of these studies. Taking this into consideration, the specific diagnostic potential of the reported miRNAs is difficult to assess and the reported correlations are only preliminary.

Overall, the limited number of reports weakly supports the potential of these circulating miRNAs as biomarkers for HCM, ACM, and DCM and additional studies involving patients affected with different cardiac disorders should be performed to firmly assess the specificity of these miRNAs for these disorders.

### Correlation between miRNA profile and cardiac abnormalities

Another aspect regards the correlation between miRNA expression and pathological cardiac features. Interstitial or replacement cardiac fibrosis is often associated with heart disease, even in the early stages, and it implies progression to end-stage forms and sudden death [45]. For several of the circulating miRNAs detected in this systematic review, the diagnostic potential correlates with mechanistic data from experimental studies performed in heart samples or in vitro models. Fibrosis was associated with circulating miR-29a-3p and miR-221 overexpression in HCM [25, 27]. Notably, cardiac miR-29a-3p was found to be dysregulated cardiomyopathies characterized by extracellular matrix remodeling, suggesting their implication in fibrosis-related pathways [46]. In ACM, circulating miR-185-5p was found upregulated in two studies involving ACM patients, but no phenotypic correlation was performed [31, 33]. Interestingly, studies on human and murine hearts linked cardiac miR-185-5p to desmosomal alterations and pro-fibrotic signaling, supporting its association with ACM [47]. Moreover, three circulating miRNAs (miR-29a-3p, miR-221, miR-199a-5p) were associated with cardiac hypertrophy, a hallmark of HCM. Of these, only miR-29a-3p positively correlated with this feature in two distinct studies on HCM patients [24, 27]. Interestingly, in Derda et al. [24], this miRNA succeeded in discriminating HOCM and HNCM. According to this finding, miR-29a-3p was related to the hypertrophic response in in vitro studies [41, 42] and it was found upregulated in the serum of 20 HCM patients, although no AUC was reported [44]. Interestingly, cardiac miR-221 and miR-199a-5p also showed roles in hypertrophic signaling pathways [48, 49].

For other candidates, such as miR-3135b and miR-5571-5p (AUC > 0.90), mechanistic evidence is still limited. This discrepancy underlines the need to prioritize those candidates showing both diagnostic and mechanistic evidence. Overall, whether, besides the tissue miRNAs, also the corresponding circulating miRNAs play an important role in cardiac phenotypes is not clear. Thus, while a correlation between diagnostic performance and biological plausibility is emerging, the mechanistic interpretation of the role of circulating miRNAs in disease-specific pathways remains preliminary and requires dedicated studies.

### Correlation between miRNA profile and genotype

HCM, ACM, and DCM are inherited disorders with incomplete penetrance and variable expressivity. Therefore, the genetic background alone is not sufficient to cause fully penetrant disease and needs to be integrated with efficient predictors. While circulating miRNA profiling holds great interest to improve patient stratification, in this systematic review, only seven miRNAs significantly correlated with genotype in affected individuals (miR-499a-5p, miR-155-5p, miR-29a-3p, let-7a-5p, miR-142-3p, miR-145-5p, miR-454-3p). For HCM, MYH7 variants were found in association with increased levels of circulating miR-499-5p (AUC = 0.95) and miR-29a-3p, while MYBPC3 haploinsufficiency was linked to reduced miR-155-5p levels [24]. However, in both cases, most mechanistic data derive from indirect evidence and do not establish a causal pathway [26]. In DCM, LMNA-related variations have been statistically linked to let-7a-5p, miR-142-3p, miR-145-5p, and miR-454-3p, but biological data remain scarce and largely extrapolated from other lamin-related conditions [50]. Taken together, while some genotype-miRNA associations emerged, biological interpretation is still speculative due to the lack of mechanistic studies in circulating samples. The correlation between miRNA profile and genotype is also limited due to the low amount of studies taking this aspect into account or including a relevant number of individuals to reach the proper statistical power. The assessment of the expression of the above-mentioned seven miRNAs in carriers of pathogenic variants in the same genes and showing milder phenotypes could help not only evaluate their specificity and sensitivity, but also study the role of these non-coding RNAs as potential modifiers. In this context, the study of Toro et al. [37] encompassed patients with both familial and idiopathic forms of DCM. Intriguingly, dysregulated circulating miRNAs were detected only in the plasma of individuals carrying pathogenic variants in LMNA, even among those who were asymptomatic. In line, Bueno-Marinas et al. found overlapping miRNA dysregulation in both ACM patients and asymptomatic relative carriers [34]. This indicates a potential influence of the genetic background on circulating miRNA expression. Therefore, further investigation into this aspect holds promise for gaining novel insights into pathogenic mechanisms and the development of innovative therapeutic strategies.

### Study limitations

Most limitations of this review reflect the current state of evidence in the field. Available studies are few, methodologically heterogeneous (particularly regarding sample types and normalization strategies), and rarely replicated in independent cohorts, resulting in low to very low certainty of evidence. In addition, the absence of longitudinal data limits conclusions regarding prognostic value. Finally, another limitation regards the restriction of eligible studies to those performing ROC analysis; while this prioritized diagnostic relevance, it reduced the overall pool of analyzed data.

Consequently, the diagnostic and mechanistic conclusions drawn here should be considered preliminary and require validation in larger, independent cohorts using standardized pre-analytical and analytical procedures.

## Supplementary Information

Supplementary Material 1. Supplementary file 1: Data extraction sheet.

Supplementary Material 2. Supplementary Figure S1: Risk of bias and quality appraisal.

## References

[1] Elliott P, Andersson B, Arbustini E, Bilinska Z, Cecchi F, Charron P, et al. Classification of the cardiomyopathies: a position statement from the European Society Of Cardiology Working Group on Myocardial and Pericardial Diseases. Eur Heart J. 2008;29(2):270–6. 17916581 10.1093/eurheartj/ehm342

[2] Prinz C, Farr M, Hering D, Horstkotte D, Faber L. The diagnosis and treatment of hypertrophic cardiomyopathy. Dtsch Arztebl Int. 2011;108(13):209–15. 21505608 10.3238/arztebl.2011.0209 PMC3078548

[3] McKenna WJ, Thiene G, Nava A, Fontaliran F, Blomstrom-Lundqvist C, Fontaine G, et al. Diagnosis of arrhythmogenic right ventricular dysplasia/cardiomyopathy. Task Force of the Working Group Myocardial and Pericardial Disease of the European Society of Cardiology and of the Scientific Council on Cardiomyopathies of the International Society and Federation of Cardiology. Brit Heart J. 1994;71(3):215–8. 8142187 10.1136/hrt.71.3.215 PMC483655

[4] Thiene G, Nava A, Corrado D, Rossi L, Pennelli N. Right ventricular cardiomyopathy and sudden death in young people. N Engl J Med. 1988;318(3):129–33. 3336399 10.1056/NEJM198801213180301

[5] Corrado D, Basso C, Judge DP. Arrhythmogenic cardiomyopathy. Circ Res. 2017;121(7):784–802. 28912183 10.1161/CIRCRESAHA.117.309345

[6] Corrado D, Link MS, Calkins H. Arrhythmogenic right ventricular cardiomyopathy. N Engl J Med. 2017;376(15):1489–90. 28402769 10.1056/NEJMc1701400

[7] Lannou S, Mansencal N, Couchoud C, Lassalle M, Dubourg O, Stengel B, et al. The public health burden of cardiomyopathies: insights from a nationwide inpatient study. J Clin Med. 2020;9(4):920. 10.3390/jcm9040920 PMC7230913 32230881

[8] Moons KG. Criteria for scientific evaluation of novel markers: a perspective. Clin Chem. 2010;56(4):537–41. 20133890 10.1373/clinchem.2009.134155

[9] O’Brien J, Hayder H, Zayed Y, Peng C. Overview of MicroRNA biogenesis mechanisms of actions, and circulation. Front Endocrinol. 2018;9:402. 10.3389/fendo.2018.00402 PMC6085463 30123182

[10] Divakaran V, Mann DL. The emerging role of microRNAs in cardiac remodeling and heart failure. Circ Res. 2008;103(10):1072–83. 18988904 10.1161/CIRCRESAHA.108.183087 PMC3982911

[11] Samanta S, Balasubramanian S, Rajasingh S, Patel U, Dhanasekaran A, Dawn B, et al. MicroRNA: a new therapeutic strategy for cardiovascular diseases. Trends Cardiovasc Med. 2016;26:407. 27013138 10.1016/j.tcm.2016.02.004 PMC4912418

[12] Alcalde M, Toro R, Bonet F, Cordoba-Caballero JC, Martínez-Barrios E, Ranea JA, et al. Role of microRNAs in arrhythmogenic cardiomyopathy: translation as biomarkers into clinical practice. Transl Res. 2023;12:11. 10.1016/j.trsl.2023.04.003 37105319

[13] Parvan R, Becker V, Hosseinpour M, Moradi Y, Louch WE, Cataliotti A, et al. Prognostic and predictive microRNA panels for heart failure patients with reduced or preserved ejection fraction: a meta-analysis of Kaplan–Meier-based individual patient data. BMC Med. 2025;23:409. 10.1186/s12916-025-04238-0 PMC12235814 40624658

[14] Morley-Smith AC, Mills A, Jacobs S, Meyns B, Rega F, Simon AR, et al. Circulating microRNAs for predicting and monitoring response to mechanical circulatory support from a left ventricular assist device. Eur J Heart Fail. 2014;16:871–9. 24961598 10.1002/ejhf.116 PMC4145708

[15] Marfella R, Di Filippo C, Potenza N, Sardu C, Rizzo MR, Siniscalchi M, et al. Circulating microRNA changes in heart failure patients treated with cardiac resynchronization therapy: responders vs non-responders. Eur J Heart Fail. 2013;15:1277–88. 23736534 10.1093/eurjhf/hft088

[16] Scolari FL, Faganello LS, Garbin HI, Piva e Mattos B, Biolo A. A systematic review of microRNAs in patients with hypertrophic cardiomyopathy. Int J Cardiol. 2021;327:146–54. 10.1016/j.ijcard.2020.11.004 33212095

[17] Chiti E, Paolo MD, Turillazzi E, Rocchi A. MicroRNAs in hypertrophic, arrhythmogenic and dilated cardiomyopathy. Diagnostics. 2021;11(9):1720. 34574061 10.3390/diagnostics11091720 PMC8469137

[18] Mylavarapu M, Kodali LSM, Vempati R, Nagarajan JS, Vyas A, Desai R. Circulating microRNAs in predicting fibrosis in hypertrophic cardiomyopathy: a systematic review. World J Cardiol. 2025;17(5):106123. 40496394 10.4330/wjc.v17.i5.106123 PMC12146958

[19] Page MJ, McKenzie JE, Bossuyt PM, Boutron I, Hoffmann TC, Mulrow CD, et al. The PRISMA 2020 statement: an updated guideline for reporting systematic reviews. BMJ. 2021;372:n71. 33782057 10.1136/bmj.n71 PMC8005924

[20] Booth A, Clarke M, Dooley G, Ghersi D, Moher D, Petticrew M, et al. The nuts and bolts of PROSPERO: an international prospective register of systematic reviews. System Rev. 2012;1:2. 22587842 10.1186/2046-4053-1-2 PMC3348673

[21] McGowan J, Sampson M, Salzwedel DM, Cogo E, Foerster V, Lefebvre C. PRESS peer review of electronic search strategies: 2015 guideline statement. J Clin Epidemiol. 2016;75:40–6. 27005575 10.1016/j.jclinepi.2016.01.021

[22] Ouzzani M, Hammady H, Fedorowicz Z, Elmagarmid A. Rayyan — a web and mobile app for systematic reviews. System Rev. 2016;5:210. 27919275 10.1186/s13643-016-0384-4 PMC5139140

[23] Whiting PF, Rutjes AWS, Westwood ME, Mallett S, Deeks JJ, Reitsma JB, et al. QUADAS-2: a revised tool for the quality assessment of diagnostic accuracy studies. Ann Intern Med. 2011;155(8):529–36. 22007046 10.7326/0003-4819-155-8-201110180-00009

[24] Derda AA, Thum S, Lorenzen JM, Bavendiek U, Heineke J, Keyser B, et al. Blood-based microRNA signatures differentiate various forms of cardiac hypertrophy. Int J Cardiol. 2015;196:115–22. 26086795 10.1016/j.ijcard.2015.05.185 PMC4936391

[25] Huang D, Chen Z, Wang J, Chen Y, Liu D, Lin K. MicroRNA-221 is a potential biomarker of myocardial hypertrophy and fibrosis in hypertrophic obstructive cardiomyopathy. Biosci Rep. 2020;40(1):BSR20191234. 10.1042/BSR20191234 PMC6954366 31868204

[26] Baulina N, Pisklova M, Kiselev I, Chumakova O, Zateyshchikov D, Favorova O. Circulating miR-499a-5p is a potential biomarker of MYH7-associated hypertrophic cardiomyopathy. Int J Mol Sci. 2022;23(7):3791. 10.3390/ijms23073791 PMC8998764 35409153

[27] Roncarati R, Anselmi CV, Losi MA, Papa L, Cavarretta E, Martins PD, et al. Circulating miR-29a, among other up-regulated MicroRNAs, is the only biomarker for both hypertrophy and fibrosis in patients with hypertrophic cardiomyopathy. J Am Coll Cardiol. 2014;63(9):920–7. 24161319 10.1016/j.jacc.2013.09.041

[28] Lombardi M, Lazzeroni D, Benedetti G, Bertoli G, Lazarevic D, Riba M, et al. Plasmatic and myocardial microRNA profiles in patients with Hypertrophic Cardiomyopathy. Clin Transl Med. 2021;11(7):e435. 34323407 10.1002/ctm2.435 PMC8287979

[29] Ntelios D, Efthimiadis G, Zegkos T, Didagelos M, Katopodi T, Meditskou S, et al. Correlation of miR-146a-5p plasma levels and rs2910164 polymorphism with left ventricle outflow tract obstruction in hypertrophic cardiomyopathy. Hellenic J Cardiol: HJC = Hellenike kardiologike epitheorese. 2021;62(5):349–354. 10.1016/j.hjc.2020.04.015 32389629

[30] Guo L, Cai Y, Wang B, Zhang F, Zhao H, Liu L, et al. Characterization of the circulating transcriptome expression profile and identification of novel miRNA biomarkers in hypertrophic cardiomyopathy. Eur J Med Res. 2023;28(1):205. 37391825 10.1186/s40001-023-01159-7 PMC10314611

[31] Yamada S, Hsiao YW, Chang SL, Lin YJ, Lo LW, Chung FP, et al. Circulating microRNAs in arrhythmogenic right ventricular cardiomyopathy with ventricular arrhythmia. Europace. 2018;20(Fi1):f37-f45. 10.1093/europace/eux289 29036525

[32] Sommariva E, D’Alessandra Y, Farina FM, Casella M, Cattaneo F, Catto V, et al. MiR-320a as a potential novel circulating biomarker of arrhythmogenic cardiomyopathy. Sci Rep. 2017;7(1):4802. 28684747 10.1038/s41598-017-05001-z PMC5500514

[33] Sacchetto C, Mohseni Z, Colpaert RMW, Vitiello L, Bortoli MD, Vonhögen IGC, et al. Circulating mir-185–5p as a potential biomarker for arrhythmogenic right ventricular cardiomyopathy. Cells. 2021;10(10):2578. 10.3390/cells10102578 PMC8533962 34685557

[34] Bueno Marinas M, Celeghin R, Cason M, Thiene G, Basso C, Pilichou K. The role of MicroRNAs in arrhythmogenic cardiomyopathy: biomarkers or innocent bystanders of disease progression? Int J Mol Sci. 2020;21(17):6434. 10.3390/ijms21176434 PMC7504260 32899376

[35] Wang H, Chen F, Tong J, Li Y, Cai J, Wang Y, et al. Circulating microRNAs as novel biomarkers for dilated cardiomyopathy. Cardiol J. 2017;24(1):65–73. 27748501 10.5603/CJ.a2016.0097

[36] Calderon-Dominguez M, Belmonte T, Ramos M, Quezada-Feijoo M, Perez-Navarro A, Alcala M, et al. Role of circulating microRNA-16–5p, a new therapeutic target for ischemic dilated cardiomyopathy. Eur J Cardio Nurs. 2020;19(1_SUPPL):S55-S55.

[37] Toro R, Blasco-Turrion S, Morales-Ponce FJ, Gonzalez P, Martinez-Camblor P, Lopez-Granados A, et al. Plasma microRNAs as biomarkers for Lamin A/C-related dilated cardiomyopathy. J Mol Med. 2018;96(8):845–56. 30008018 10.1007/s00109-018-1666-1

[38] Aleshcheva G, Pietsch H, Escher F, Schultheiss HP. MicroRNA profiling as a novel diagnostic tool for identification of patients with inflammatory and/or virally induced cardiomyopathies. ESC Heart Failure. 2021;8(1):408–22. 33215881 10.1002/ehf2.13090 PMC7835602

[39] Cheng X, Jian D, Xing J, Liu C, Liu Y, Cui C, et al. Circulating cardiac MicroRNAs safeguard against dilated cardiomyopathy. Clin Transl Med. 2023;13(5):e1258. 37138538 10.1002/ctm2.1258 PMC10157268

[40] Fan KL, Zhang HF, Shen J, Zhang Q, Li XL. Circulating microRNAs levels in Chinese heart failure patients caused by dilated cardiomyopathy. Indian Heart J. 2013;65(1):12–6. 23438607 10.1016/j.ihj.2012.12.022 PMC3860780

[41] Fayez AG, Esmaiel NN, Salem SM, Ashaat EA, El-Saiedi SA, El Ruby MO. miR-454–3p and miR-194–5p targeting cardiac sarcolemma ion exchange transcripts are potential noninvasive diagnostic biomarkers for childhood dilated cardiomyopathy in Egyptian patients. Egypt Heart J: (EHJ). 2022;74(1):65. 10.1186/s43044-022-00300-x PMC9458794 36076093

[42] Jiao M, You H, Wang Z, Gu Y, Wang X, Liang Y, et al. Diagnosis of dilated cardiomyopathy in children based on microRNA sequencing technology. Chin J Appl Clin Pedia. 2020;35(13):982–7.

[43] Maron BJ, Maron MS. Hypertrophic cardiomyopathy. Lancet (London, England). 2013;381(9862):242–55. 22874472 10.1016/S0140-6736(12)60397-3

[44] Sonsöz MR, Yilmaz M, Cevik E, Orta H, Bilge AK, Elitok A, et al. Circulating levels of MicroRNAs in hypertrophic cardiomyopathy: the relationship with left ventricular hypertrophy, left atrial dilatation and ventricular depolarisation-repolarisation parameters. Heart Lung Circ. 2022;31(2):199–206. 34088630 10.1016/j.hlc.2021.04.019

[45] Marian AJ, Braunwald E. Hypertrophic cardiomyopathy: genetics, pathogenesis, clinical manifestations, diagnosis, and therapy. Circ Res. 2017;121(7):749–70. 28912181 10.1161/CIRCRESAHA.117.311059 PMC5654557

[46] Hsu CH, Liu IF, Kuo HF, Li CY, Lian WS, Chang CY, et al. miR-29a-3p/THBS2 axis regulates PAH-induced cardiac fibrosis. Int J Mol Sci. 2021;22(19):10574. 34638915 10.3390/ijms221910574 PMC8509017

[47] Lin R, Rahtu-Korpela L, Szabo Z, Kemppi A, Skarp S, Kiviniemi AM, et al. MiR-185-5p regulates the development of myocardial fibrosis. J Mol Cell Cardiol. 2022;165:130–40. 34973276 10.1016/j.yjmcc.2021.12.011

[48] Kakimoto Y, Tanaka M, Hayashi H, Yokoyama K, Osawa M. Overexpression of miR-221 in sudden death with cardiac hypertrophy patients. Heliyon. 2018;4(6):e00639. 30009269 10.1016/j.heliyon.2018.e00639 PMC6041564

[49] Zhang H, Li S, Zhou Q, Sun Q, Shen S, Zhou Y, et al. Qiliqiangxin attenuates phenylephrine-induced cardiac hypertrophy through downregulation of miR-199a-5p. Cell Physiol Biochem. 2016;38(5):1743–51. 27161004 10.1159/000443113

[50] Fenzl FQ, Lederer EM, Brumma L, Krüger P, Schroll M, Wilming F, et al. Deregulated miR-145 and miR-27b in Hutchinson-Gilford progeria syndrome: implications for adipogenesis. Aging. 2025;17(9):2278–311. 40874920 10.18632/aging.206309 PMC12517220

